# Individual experience affects host choice in malaria vector mosquitoes

**DOI:** 10.1186/1756-3305-7-249

**Published:** 2014-05-29

**Authors:** Amélie Vantaux, Thierry Lefèvre, Kounbrobr Roch Dabiré, Anna Cohuet

**Affiliations:** 1 UMR MIVEGEC (IRD 224 - CNRS 5290 - UM1 - UM2), 911 avenue Agropolis, BP 64501, 34394 Montpellier cedex 5, France; 2 Institut de Recherche en Sciences de la Santé, 01 BP 171 Bobo Dioulasso, Burkina Faso; 3 Centre Muraz, Bobo Dioulasso, Burkina Faso

**Keywords:** Experience, Host choice, Feeding behaviour, Mosquitoes, *Anopheles coluzzii*, *Anopheles gambiae* M form, Malaria, Vector, Transmission

## Abstract

**Background:**

Despite epidemiological importance, few studies have explored whether individual experience and learning could affect the vertebrate host choice of mosquito disease vectors. Here, we investigated whether a first successful blood meal can modulate mosquito preference during a second blood meal.

**Methods:**

In no-choice situations, females of the mosquito *Anopheles coluzzii*, one of the primary African malaria vectors, were first allowed to feed on either human, rabbit or guinea pig. Four days later in dual-choice situations, the same mosquitoes were allowed to choose between the two uncommon hosts, rabbit and guinea pig, as a source of blood. ELISA assays were then used to determine which host mosquitoes fed on.

**Results:**

Our results indicate that, overall, mosquitoes preferred to feed on rabbit over guinea pig and that the nature of the first blood meal had a significant impact on the mosquito host choice during the second blood meal. Compared to mosquitoes that previously fed on guinea pigs or humans, mosquitoes that fed on rabbits were less likely to choose this host species during a second exposition. The decreased preference for rabbit was observed four days after mosquitoes were first exposed to this host, suggesting that the effect lasts at least the duration of a gonotrophic cycle. Furthermore, this effect was observed after only one successful blood meal. Fitness measurements on mosquitoes fed on the three different vertebrate hosts showed that the origin of the blood meal affected mosquito longevity but not fecundity. In particular, human-fed mosquitoes lived longer than guinea pig-fed or rabbit-fed mosquitoes.

**Conclusions:**

Our study demonstrates that individual experience affects host choice in this mosquito species and might have strong repercussions on biting patterns in natural conditions and hence on malaria transmission.

## Background

The decisions that animals make when selecting a resource such as habitat, breeding sites, food or host affect both the ecological interactions in which they participate and the selective pressures that shape their life-history traits. Consequently, the ability to change behaviour after experience is adaptive to adjust to environmental unpredictability [1]. The effects of individual experience in insects can be observed in many situations, from natal habitat preference induction [2], to foraging [3], mating [4], oviposition [5], predator avoidance [6], medication against parasites [7] or social interactions [8]. Learning can be defined as one complex form of individual experience, in which behavioural changes are linked to cognitive and memorization processes e.g. conditioning, habituation or sensitization; [1,9-11], while other individual experiences can be the result of different physiological processes (e.g. motor/sensory fatigue).

Despite their medical significance as vectors of dangerous human diseases such as malaria, yellow fever, dengue fever, and filariasis, few studies have examined the effect of individual experience and learning in mosquitoes [12,13]. Aside from a field study describing the tendency of *Culex* mosquitoes to feed on vertebrate host species that they had been previously attracted to [14], most current evidence for the occurrence of learning and memory in mosquito vectors comes from recent laboratory controlled-experiments in which insects were conditioned to associate colours or odours with sugar- or blood-meals [15-19]. These studies are an important step forward. They not only highlight that mosquito disease-vectors possess the cognitive machinery required to associate environmental cues and resources, they also suggest that they may learn about stimuli emitted by their vertebrate hosts. This possibility may have important implications.

First, the potential ability of mosquitoes to respond adaptively to host choice in natural conditions would increase our basic understanding of their blood-feeding behaviour. Until now, the factors proposed to influence mosquito host species choice have included mosquito genetic background, host availability and accessibility, blood nutritional value, energetic costs of digestion, and host defensive behaviours [20]. Exploring the effects of past experience on mosquito host choice may therefore reveal that individual experience should be added to the list of factors that modulate the contact rate between mosquito vectors and their vertebrate hosts in the field.

Second and most importantly, individual experience effects on host preference may have crucial epidemiological consequences. At the host inter-specific level, a general expectation is that mosquito individual experience will result in repeated feeding on the same host species, thereby increasing the risk of disease transmission among host individuals of the same species (assuming the disease-causing agent is host-specific such as e.g. the malaria parasite *Plasmodium falciparum*, [12]).

The current study examined whether *An. coluzzii* formerly *Anopheles gambiae* M molecular form, [21], a major vector of the deadliest human malaria parasite *P. falciparum*, can preferentially return to feed on host species from which past blood-feeding was successful. Although this species is considered a highly anthropophilic mosquito [22-24], they can feed on a wide range of other vertebrates in nature e.g. [25-31]. The learning and memory capacities of this species have recently been uncovered [19]. Using membrane-feeding assays, the authors showed that *An. coluzzii* females were able to rapidly associate a visual or an olfactory stimulus with a positive or negative reinforcing stimulus (blood meal quality), with a maximum memory retention of 72 h [19]. This study generated a significant body of knowledge on the learning and memory capacities of this important malaria vector, but many questions remain. In particular, could feeding completion on a given host species result in reinforcement, such that this becomes the preferred host at the next blood-meal?

To address this question, female *An. coluzzii* were first given the opportunity to feed on two uncommon hosts, either rabbits or guinea pigs. We used an experimental setting designed to accommodate the whole host body odour as well as defensive behaviours as sources of stimuli. Other mosquitoes were fed with human blood and used as controls. Four-days later, at the next gonotrophic cycle, we allowed the mosquitoes to choose between rabbit and guinea pig. We predicted that mosquitoes should display preference for the host species from which they previously obtained a successful blood-meal. Because both individual experience processes and/or genetic predisposition could explain the predicted pattern, we also verified that mosquito offspring did not display a similar level of host preference to their mothers. Finally, to link mosquito trophic preferences to fitness benefits, we measured mosquito longevity and fecundity after feeding on the three different vertebrate hosts used in this study. We show that the first blood-meal source influences the host species selected by *An. coluzzii* 4 days-later, but in unexpected ways.

## Methods

### Mosquitoes

*Anopheles* mosquitoes were collected at larval stages in the Vallée du Kou (Southwestern Burkina Faso) during June-July 2012. The larvae were bred in the laboratory using water from the collection sites and received *ad libitum* Tetramin® food. After emergence adults had access to a 5% glucose solution on cotton wool pads until 3 to 5 days old. A representative sample of 118 adult females was identified by species diagnostic PCR [32]. In agreement with previous studies in the area [26,33-35], all genotyped individuals belonged to the species *An. coluzzii*, suggesting that almost all, if not all, mosquitoes included in this study belonged to this species. To ensure their willingness to feed, mosquitoes were provided only water for 24 h prior to access to a blood meal.

### Experimental design

#### *Individual experience test*

In no choice situations, 100 female mosquitoes were exposed to their first host either consisting of a rabbit, a guinea pig or a human (used as a control). Rabbits and guinea pigs were placed in individual cages of 60 × 40 × 40 cm covered with a mesh and were provided with similar amounts of water and food. Mosquitoes were then released in the cages overnight such that total host body odour (breath, skin emanations, animal excrements) as well as defensive behaviours were used as a source of stimuli. Control mosquitoes were fed using direct feeding assays, whereby the arm of one of the experimenters is placed for one hour over a piece of mesh covering a cup full of mosquitoes. In the morning, blood-fed female mosquitoes were collected with a mouth aspirator and transferred to cages (20×20×20 cm) with *ad libitum* water and 5% glucose solution on cotton pads. Unfed females were discarded. Oviposition dishes consisting of water cups were placed in each cage.

Four days after their first blood meal, female mosquitoes were coloured with one of three different coloured powders (Luminous Powder Kit, BioQuip), corresponding to the host species they had previously fed on. The matching between host species and colours was switched between replicates. The mosquitoes were then released overnight in a cage containing one rabbit and one guinea pig. A barrier of half the height of the cage was set-up in the middle of the cage such that the rabbit and the guinea pig were separated but the mosquitoes were free to fly from one host to another. The animals received identical qualities and quantities of food and water. In the morning, blood-fed females were collected with a mouth aspirator and frozen at -20°C. Unfed females were discarded.

The origin of mosquito blood-meals was determined using enzyme-linked immunosorbent assay (ELISA). The protocol was adapted from previous blood meal identification tests [36] by using 1/2000 dilutions of anti-guinea pig and anti rabbit IgG (Sigma-Aldrich).

The individual experience test (i.e. no-choice assay followed by dual-choice assay) was repeated 12 times, using three rabbits, three guinea pigs, and two different human bloods and a total of 3600 mosquitoes. We tested all possible combinations between each rabbit and guinea pig (i.e. 9 combinations) at least once, and 3 combinations were tested twice.

#### *Offspring preference*

Because increased mosquito preference for the host species previously used as a source of blood-meal may result from effects of past experience (e.g., learned preference) or from intrinsic behaviours (genetic preference) [13], we also measured offspring host choice. The water cups provided to the F0 females for oviposition were collected and hatched larvae were bred in the same conditions as the parental generation. Once they were 3 to 5 days old, female mosquitoes were coloured with three different coloured powders corresponding to the host species their mothers fed on. They were then pooled together and released overnight in a cage containing the same rabbit and guinea pig their mothers fed on. In the morning, blood fed females were collected with a mouth aspirator and frozen at -20°C. The blood meal origin was determined using ELISA assays as described above. We tested 6 different combinations using 741 mosquitoes. The correspondence between hosts and colours were changed between combinations.

#### *Fitness experiment*

In order to link host species preferences to fitness benefits, we compared the longevity and fecundity of mosquitoes fed on the three different vertebrate hosts. Field mosquitoes were reared as previously described. Three to five day old female mosquitoes were transferred in cups of 30 individuals covered by a piece of mesh. Mosquitoes were fed using direct feeding assays. Rabbits and guinea pigs were shaved on 5×5 cm area of their back to facilitate feeding. The cups were maintained by the experimenters to allow mosquito feeding on the respective host for 30 minutes. All fed mosquitoes were transferred into cages (20×20×20 cm). Mosquitoes were checked daily for mortality. Mosquito fecundity was measured from females that received a second blood meal from the same host and in the same conditions 4 days later in order to ensure sufficient egg-laying females [37-42]. Two days after their second blood meal, females were placed in individual plastic cups with an oviposition dish and followed until death. The presence and the numbers of eggs were recorded. Four guinea pigs, five rabbits, six humans and a total of 336 mosquitoes (about 22 mosquitoes per individual host) were used for the survivorship assay. Five guinea pigs, four rabbits, six humans, and a total of 152 mosquitoes (about 10 per individual hosts) were used for the fecundity assay.

### Statistical analyses

We observed only thirteen mixed blood meals (individuals that fed on both rabbit and guinea pig) in both feeding choice tests (individual experience test and offspring preference), thus these samples were not included in the analyses. We tested the effect of past experience (i.e. the host species used in the no-choice assay for the individual experience test, or the host species mothers fed on for the offspring preference experiment) on mosquito host species choice using a Generalized Linear Mixed Model (GLMM) with a binomial error structure and logit link function. A binomial GLMM was also used to examine the effect of host species on the proportion of engorged mosquitoes (feeding rate) during the no-choice and dual-choice assays. In these GLMMs, host species was coded as a fixed categorical factor, and the host individual identity (no-choice assay), or the combination of host individuals (dual-choice assay) was coded as a random factor. We also verified whether the proportions (p) of mosquitoes fed on rabbit (or guinea pig) during the dual-choice assays were compatible with a random choice (null hypothesis: p = 0.5) or whether mosquitoes displayed a statistically significant preference (H1: p ≠ 0.5). Chi-square post hoc tests were carried out to assess differences between hosts, and Bonferroni corrections were applied for multiple comparisons.

Survival curves of mosquitoes fed on the three different vertebrate hosts were compared using a Cox proportional hazards model, and fecundity was compared using a GLM with a binomial distribution for the presence of eggs and a quasipoisson distribution for the number of eggs laid.

The significance of the explanatory variable was established using a likelihood ratio test (LRT). Analyses used lme4, MASS, survival and multcomp packages in R v. 2.15.0 [43-47].

### Ethical notes

The rabbits and guinea pigs were used only to feed the mosquitoes from the study. After the study, the rabbits and guinea pigs were euthanized. All mosquitoes were killed by putting them at -20°C for 30 min. All humans provided informed written consent before participation. Ethical approval was obtained from the Centre Muraz Institutional Ethics Committee under the ethical clearance number A003-2012/CE-CM, and all experiments carried out at the IRSS are under the Animal Welfare Assurance A5926-01.

## Results

**Figure 1:**
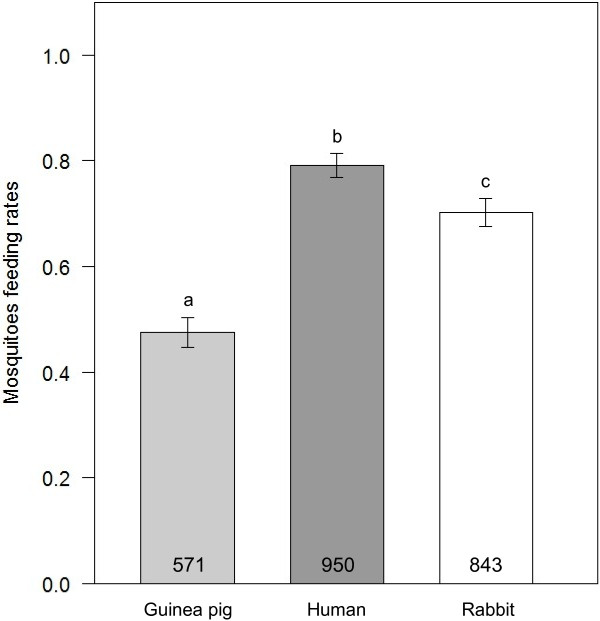
**Feeding rates of *Anopheles coluzzii *mosquitoes in the no choice assay.** Error bars show 95% confidence intervals. Different letters indicate significant differences (Post hoc chi-square tests with a Bonferroni correction, P < 0.05).

**Figure 2:**
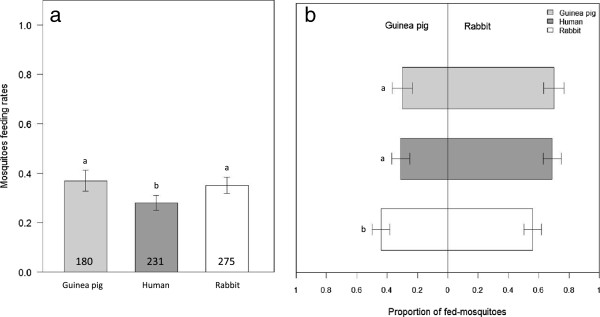
**Feeding rates (a) and preference (b) of *Anopheles coluzzii *mosquitoes in the dual-choice assay.** Grey scales indicate the host they fed on during their first blood meal. Error bars show 95% confidence limits. Different letters indicate significant differences (**a**- Post hoc chi-square tests with a Bonferroni correction, P < 0.05, **b**- Post hoc chi-square tests with a Bonferroni correction, P < 0.05).

The overall proportion of engorged mosquitoes (feeding rate) in the no-choice assay (first blood-meal) was 65.7 ± 2% (proportion ± 95% Wald’s type confidence interval). There was a significant effect of host species on feeding rate, with higher proportions of human-fed (79.17 ± 2%) and rabbit-fed mosquitoes (70.25 ± 2%) than guinea pig-fed mosquitoes (47.58 ± 3%, GLMM, *N* = 3600, *X*^2^_2_ = 7.01, *P* = 0.03, Figure 1). The overall proportion of engorged mosquitoes in the dual-choice assay (second blood-meal, 32.1 ± 2%) was lower than in the no-choice assay (Chi square test, *X*^2^_1_ = 597.3, *P* < 0.001, Figure 1 and Figure 2a). The host species used for the first blood-meal influenced mosquito feeding rate during the dual-choice assay, with rabbit-fed and guinea pig-fed mosquitoes being more likely to feed than human-fed mosquitoes (35.07 ± 3%, 36.96 ± 4%, 28.03 ± 3%, respectively; GLMM, *N* = 2095, *X*^2^_2_ = 28.4, *P* < 0.001, Figure 2a). When compared to the first blood-meal, the feeding rate of human-fed mosquitoes during the dual-choice assay decreased by almost 3 fold whereas that of rabbit- and guinea pig-fed mosquitoes decreased by 2 and 1.25 fold, respectively.

Regardless of the host species on which they previously fed, mosquitoes displayed an overall preference for rabbit over guinea pig (GLM, *N* = 686, Odds Ratio (OR) = 1.77, 95% confidence interval (CI) = 1.52-2.07, *P* < 0.001). This attraction was not enhanced by previous experience. The nature of the first blood meal influenced the host choice at second gonotrophic cycle: compared to human-fed and guinea pig-fed mosquitoes, rabbit-fed mosquitoes showed a decreased preference for rabbit (GLMM, *N* = 686, *X*^2^_2_ = 13.5, *P* < 0.001, Figure 2b).

Offspring mosquitoes also displayed an overall preference for rabbit over guinea pig (GLM, N = 290, OR = 1.48, CI = 1.87-1.69, *P* = 0.001). The origin of the blood meal taken by the mothers did not significantly influence the blood meal choice of their progeny (GLMM, *N* = 290, *X*^2^_2_ = 0.38, *P* = 0.83).

**Figure 3:**
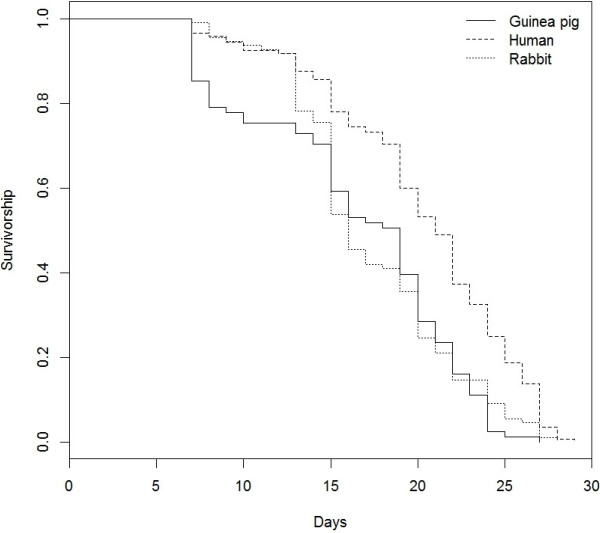
**Survival curves of *Anopheles coluzzii *** **mosquitoes fed on three different vertebrate hosts.**

Mosquito survival was significantly affected by the blood meal type (Cox model, *N* = 336, *X*^2^_2_ = 32.15, *P* < 0.001). In particular, human-fed mosquitoes lived longer than rabbit-fed or guinea pig-fed mosquitoes (Figure 3, mean longevity ± SE: 20.26 ± 0.45, 17.33 ± 0.45 and 16.49 ± 0.66 days, respectively). However, blood meal type had no effect on either the probability of laying eggs (proportion of egg-laying females = 51 ± 10%, 38 ± 15%, 42 ± 20% respectively for human-fed, rabbit-fed and guinea-pig-fed mosquitoes, GLM, *N* = 152, *X*^2^_2_ = 1.83, *P* = 0.4) nor the number of eggs laid (mean ± SE: 74.71 ± 5.72, 66.5 ± 8.36 and 78.9 ± 11.2 eggs, respectively, GLM, *N* = 70, X^2^_2_ = 15.2, *P* = 0.66). Together, these findings suggest that mosquito fecundity was not affected by the host species used for blood-feeding.

## Discussion

We found that the previous feeding experience encountered by *An. coluzzii* influenced its vertebrate host preference but in unpredicted ways. In particular, rabbit-fed mosquitoes displayed a decreased preference for rabbit compared to human-fed or guinea pig-fed mosquitoes. Since overall mosquitoes preferred to feed on rabbit over guinea pig, this implies that fewer rabbit-fed mosquitoes would try to feed on it for the next blood meal even if the choice is with an intrinsically less preferred host.

In experiments testing the effect of experience on mosquito behaviour, care must be taken to not interpret selection of best genetically adapted individuals among a polymorphic population as behavioural plasticity [13]. Here, we observed a decreased preference, which allows excluding that we artificially selected for mosquitoes having an innate preference for a given vertebrate host, otherwise we would have observed the reverse pattern (i. e. an increased preference for rabbit or guinea pig for the mosquitoes that previously fed on rabbit or guinea pig, respectively). In addition, we carried out the same choice assay on mosquito progeny and, as expected, did not find an increased preference for the host their mothers fed on.

In no choice assays, mosquitoes displayed higher feeding rates on humans and rabbits than on guinea pigs. Possible explanations include differences in size, defensive behaviours, or mosquito innate aversion due to repulsive odours emitted by this host species. We also found that mosquitoes had the highest feeding rate on humans, which is their natural preferred host [22-24]. However, this needs to be taken with caution as the way mosquitoes were exposed to a human host unlike rabbit- and guinea pig-exposed mosquitoes prevented any defensive behaviour and used an exposure time which could both have influenced human-exposed mosquitoes feeding rate.

When looking at innate preferences, *An. coluzzii* anthropophilic behaviour corresponded to a fitness benefit to feed on humans, these mosquitoes having a better survival than those feeding on rabbit or guinea pig, these two last hosts having longevity within the same range. In addition, no fecundity difference was found among mosquitoes fed on the three host species. Therefore, we did not find a positive relationship between fitness performance and preference in our setup. Since we did not allow defensive behaviours when mosquitoes were fed for measuring fitness traits, the blood quality seemed to play a minor role in fitness differences between these hosts. Nonetheless, the physiological use of the blood meals would need to be tested in other conditions (e.g. flight activity) as blood quality might affect mosquito fitness differently depending on the context [48,49]. In addition, defensive behaviour-derived mortality or limited feeding were not taken into account and may induce substantial fitness costs.

Overall, mosquito feeding rate was lower in dual choice situations than in no choice situations. Despite allowing females to lay eggs, and waiting four days before the subsequent blood meal, females might have been less motivated to bite than for their first blood-meal, and might have preferred to wait for a future and better opportunity. A second possibility could lie in the fact that, in the dual choice assays, animals were kept side by side within the same cage. This could have increased their level of stress compared to the no-choice assays and thereby increased their level of defensive behaviours. A third explanation might be that the vertebrate hosts learned to better defend themselves after the first night spent with mosquitoes.

The feeding rate in the dual choice assay was also influenced by the previous experience of the mosquito: human-fed mosquitoes were less likely to feed than rabbit- and guinea pig-fed mosquitoes when offered a choice between guinea pig and rabbit. Indeed, human-fed mosquitoes displayed the highest discrepancy in their feeding rate between the no choice and the choice assays. This suggests that, after a successful blood meal on humans, mosquitoes postponed a blood meal on uncommon and possibly less preferred hosts, such as rabbit or guinea pig in our case. The survival difference observed in the fitness experiment suggests a better nutritive value of human blood compared to guinea pig or rabbit blood, which would corroborate this hypothesis. This is of importance for the epidemiology of vector-transmitted diseases as any effect of individual experience on biting frequency may be expected to have a major impact on parasite transmission [12].

In contrast to the few existing studies on the effects of past experience on mosquito feeding behaviour [14-19], our experimental design did not use exposition to one specific odor but to the complete host’s odor as a source of stimuli. Furthermore, our design encompassed a longer behavioural sequence, from short-range location and host choice to the realized blood meal. This way, we were able to take into account host defensive behaviours, which has been shown to lower feeding rate [50-52] as well as host acceptance linked to innate host characteristics such as temperature or blood quality [20,23]. All these factors have been shown to be involved in decision processes leading to host location, recognition and acceptance [23,53].

By giving the choice to female mosquitoes four days later, we followed natural mosquito rhythm allowing completing a reproductive cycle and we were able to show that effects of individual experience can last not only for a long time but most importantly from one blood meal to another. It is worth noting that one successful feeding experience was sufficient to elicit a decreased preference for rabbit.

## Conclusions

We found that the previous feeding experience of *An. coluzzii* influenced its vertebrate host preference with rabbit-fed mosquitoes displaying a decreased preference for rabbit compared to human-fed or guinea pig-fed mosquitoes. Despite using uncommon vertebrate hosts for *An. coluzzii* mosquitoes, we demonstrated that individual experience affects its vertebrate host choice, and thus can possibly affect malaria transmission risk. Future studies, using ecologically relevant host species such as humans and cattle, are required to better assess the role that individual experience and learning may play in malaria transmission.

## Abbreviations

ELISA: Enzyme-linked immunosorbent assay; PCR: Polymerase chain reaction; GLMM: Generalized linear mixed model; GLM: Generalized linear model; IRSS: Institut de Recherche en Sciences de la Santé.

## Competing interests

The authors declare that they have no competing interest.

## Authors’ contributions

AV, TL and AC conceived and designed the experiments. AV carried out the experiments, performed the statistical analyses and drafted the manuscript. TL performed the statistical analyses. RD and AC coordinated the study. All authors read and approved the final manuscript.
